# Isolation Related to the COVID-19 Pandemic in People Suffering from Parkinson’s Disease and Activity, Self-Assessment of Physical Fitness and the Level of Affective Disorders

**DOI:** 10.3390/healthcare9111562

**Published:** 2021-11-17

**Authors:** Andrzej Knapik, Justyna Szefler-Derela, Dagmara Wasiuk-Zowada, Joanna Siuda, Ewa Krzystanek, Anna Brzęk

**Affiliations:** 1 Department of Adapted Physical Activity and Sport, School of Health Sciences in Katowice, Medical University of Silesia, 40-754 Katowice, Poland; aknapik@sum.edu.pl; 2 Department of Physiotherapy, School of Health Sciences in Katowice, Medical University of Silesia, 40-754 Katowice, Poland; jszefler@sum.edu.pl (J.S.-D.); dwasiuk@sum.edu.pl (D.W.-Z.); 3 Department of Neurology, Faculty of Medical Sciences in Katowice, Medical University of Silesia, 40-754 Katowice, Poland; jsiuda@sum.edu.pl (J.S.); ekrzystanek@sum.edu.pl (E.K.)

**Keywords:** Parkinson’s disease, COVID-19, physical activity

## Abstract

Background: Staying at home for long periods and limiting various types of activities and social contacts due to the COVID-19 pandemic may have negative consequences for health. This is especially true for people suffering from chronic diseases, in whom an appropriate level of activity and social contacts delay the progress of the disease. This group includes people diagnosed with Parkinson’s disease—PD. Aim: It was decided to investigate the effect of COVID-19 isolation related to self-assessment of physical fitness, physical activity, and the level of anxiety and depression in people with PD. Methods: The study included 30 patients diagnosed with Parkinson’s disease. We compared the results of the pre-pandemic questionnaire and the telephone interview with the same questions—after the period of isolation due to COVID-19. The questionnaire included questions about physical activity and fitness self-assessment. The level of affective disorders was tested using HADS. Results: There was a statistically significant decrease in the physical activity of the respondents after isolation related to COVID-19 (*p* < 0.05). Self-assessment of physical fitness also decreased, but the differences were not statistically significant. In the post-isolation study, only 50% of the respondents had normative values for anxiety and only 40% for depression. The analysis showed that the level of physical activity—the independent variable, explains anxiety in 30% and depression in 27%. Conclusions: Pandemic isolation has significantly reduced physical activity in PD patients. There was a certain drop in the self-esteem of physical fitness in these people. Physical fitness is an important predictor of preventing the affective disorders of anxiety and depression. The effects of isolation due to COVID-19 require further research.

## 1. Introduction

Due to the emergence of the COVID-19 pandemic in the world, many countries have introduced various types of restrictions in contacts between people. They were intended to prevent the spread of the epidemic. In Poland, as in other countries, people with COVID-19 virus presence were subjected to hospitalization when required by their health condition. The rest of the sick, as well as those living with them, were strictly forbidden to leave the house until tests showed no virus. It was also ordered that people not suffering from COVID-19 should not leave their home or limit their going out to the necessary minimum. This was especially true of people for whom COVID-19 infection was a serious threat to health and life. First of all, it was about the elderly and chronically ill. This group includes people suffering from Parkinson’s disease (PD). The isolation of these patients was especially justified due to their greater susceptibility to the virus. It is associated with both direct causes, such as stiffness of the respiratory muscles, weakened cough reflex, pre-existing dyspnea [1], and indirect ones. They can be stress, fear, or prolonged immobility [2]. The relationship between viral infections and neurodegeneration is also considered [3]. The isolation of PD patients, in addition to increasing the safety margin against SARS-CoV-2 infection, had consequences, the examination of which may constitute a premise for a possible modification of further care for PD patients.

It was decided to investigate to what extent staying at home—due to the limitations related to the pandemic, had an impact on the self-esteem of physical fitness, physical activity, and the level of anxiety and depression in people with PD.

## 2. Materials and Methods

### 2.1. Participants

All respondents were residents of the Silesian Voivodeship, Southern Poland. Thirty people were examined—12 women (40%) and 18 men (60%). The sample size was calculate using Raosft [4] with assumptions margin error 5%, confidence level 95%, from a random sample of 35 patients with PD participating in a PD support group and at the PD Association. With assumption of 35 participants, the simple size was estimated to be 32, but the study was able to screen 30 PD patients planned in the study. Participation in the study was voluntary. Age of respondents: mean: 69.73, SD = 7.91, (±95 CI: 66.78–72.69) years. These were people with PD treatment durations with means of 7.68, SD = 5.02, (±95 CI: 5.81–9.56) years. All persons were under the constant care of specialist doctors and took their medications as recommended. Neither person had DBS. Disease stage: I–III stage according to Hoehn-Yahr.

The subjects belonged to a support group that met systematically before the epidemic. These meetings were attended by neuroscientists and physiotherapists who care for PD patients. The tests were performed twice. The first study was performed a few months before the COVID-19 epidemic—as part of meetings with people suffering from PD. Then, the respondents filled in the questionnaire on their own. If necessary, the researchers provided help. The second examination was performed during the epidemic—after 90 days of the recommendations to limit leaving home. Due to limitations, they were made using the telephone interview method. The research questionnaire was then supplemented with questions related to the epidemic. Information was also obtained that none of the respondents was infected with COVID-19. 

### 2.2. Methods

The research questionnaire consisted of a metric part, where data on gender, age, and years of PD treatment were collected. The respondents were also asked about the way of living regarding running a household. This was the basis for dividing the respondents into two groups: A—people living alone (n = 6; 20% of the total), B—people living with a spouse or family (n = 24; 80%). The next questions concerned the current functional aspects without specifying a time frame: self-assessment of physical fitness (SAPF) and physical activity (PA). The statements marked by the respondents were ranked according to the intensity of the feature. Statements about SAPF include:

1. I am disabled, I need help to perform most of the activities;
2. I am not very fit, I often need help;
3. I am partially functional, I need help in carrying out only some of the activities;
4. I am fit, but some activities are difficult for me;
5. I am fully functional, I do not require any help in everyday life [5].

Statements concerning PA included: self-esteem of activity, its intensity, inactivity, and walking (frequency and time). For this purpose, an adapted part of the Baecke questionnaire was used [6,7,8]:

I. I believe that my activity, compared to other people my age, is:

1—much smaller; 2—smaller; 3—the same; 4—bigger; 5—much bigger;

II. In my spare time, I am active, exercise, and do sports:

1—never; 2—rarely; 3—sometimes; 4—often; 5—very often;

III. In my spare time I spend my time sitting or lying:

1—very often; 2—often; 3—sometimes; 4—rarely; 5—never;

IV. In my spare time I walk:

1—never; 2—rarely; 3—sometimes; 4—often; 5—very often;

V. How much time a day do you walk away from home—e.g., walking, shopping, other activities:

1—less than 5; 2—5–15 min; 3—15–30 min; 4—30–45 min; 5—over 45 min.

Based on the PA responses, the Physical Activity Index (PAI) was calculated. It was the sum of the activity response scores.

In the second study, in addition to those presented above, questions related to the COVID-19 epidemic were also asked. They concerned the number of days when the respondents did not leave the house. They were also asked to leave home before the epidemic and to leave home now. In both cases, there were three variants of the answer: 1—only when necessary; 2—rather rarely; 3—regularly.

Moreover, in the second study, the mental state of the patients was examined using The Hospital Anxiety and Depression Scale (HADS) [9]. This tool enables the monitoring of depressive and anxiety reactions in patients with somatic diseases. It is also used in patients with PD [10]. The questionnaire consists of fourteen items which were assigned a scale from 0 to 3 responses. Seven items form a subscale for anxiety, the remaining seven for depression. In both scales, points are added up. A qualitative assessment was adopted: 0–7 is the norm, 8–10 points are borderline values, and 11–21 points are disorders. The psychometric properties of HADS have been positively verified on the basis of studies from many countries, including Polish studies [11,12].

The respondents were also asked whether they had had contact with a doctor during isolation. Among the surveyed, 17 people (56.67%) sought advice by phone, and the remaining 13 people (43.33%) did not contact a doctor. All subjects continued pharmacological treatment as previously recommended. None of the patients had treatment altered during isolation.

### 2.3. Ethics Approval

The study was conducted in accordance with the provisions of the Helsinki Convention, and the Bioethics Committee of the Medical University of Silesia Katowice also expressed its approval (Decision no.: PCN/0022/KB1/104/20).

### 2.4. Statistical Analysis

Descriptive statistics were performed. Non-parametric tests were used for the comparisons. Intergroup comparisons were made using the Mann–Whitney U test. The comparison of SAPF and PAI between tests I and II was made using the sign test. The influence of selected variables on the level of anxiety and depression of the respondents was assessed by means of linear regression. Level of significance adopted: *p* < 0.05.

## 3. Results

The comparison of the age of the subjects and the years of treatment between women and men showed no differences—for age: *p* = 0.536, respectively, for treatment time: *p* = 0.380. Gender was also not a differentiating variable in either group A (Study I—SAPF: *p* = 0.683, PAI: *p* = 0.698; Study II: SAPF: *p* = 0.219, PAI: *p* = 0.487), or in group B (Study I—SAPF: *p* = 0.184, PAI: *p* = 0.566; Study II—SAPF: *p* = 0.849, PAI: *p* = 0.228). There were also no differences in anxiety and depression in Study II. Group A—anxiety: *p* = 0.817, depression: *p* = 0.481. Group B—anxiety: *p* = 0.356, depression: *p* = 0.951. This allowed treating the group as homogeneous in these respects.

The average number of days not leaving home was 42.33 (SD = 35.70). The comparison between groups A and B showed no differences: *p* = 0.4939.

The analysis of the results of studies I and II showed no differences between groups A and B, neither with regard to SAPF nor PAI. In addition, the comparison of the variables studied only in Study II showed no differences between the groups in any case. There was only a statistically significant decrease in PAI for the total number of respondents when comparing Study II with Study I (Table 1).

The comparison of the variables from Study II showed no differences between group A and group B regarding behavior and the level of anxiety and depression (Table 2).

**Figure 1:**
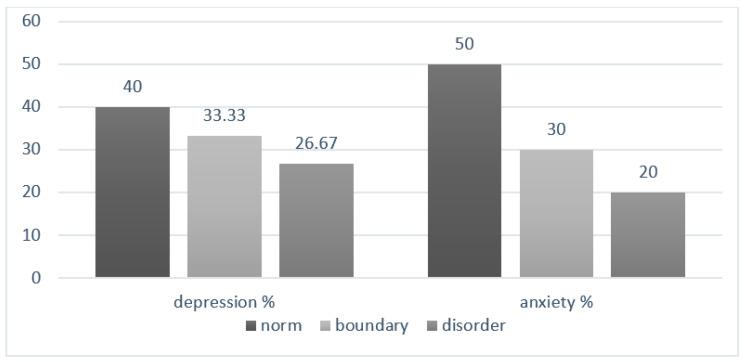
Qualitative assessment of anxiety and depression—Study II.

Qualitative analysis of the HADS questionnaire results showed high percentages of people with borderline values or with disorders. In the case of anxiety, half of the respondents had normative values and in the case of depression only 40% (Figure 1).

When examining the influence of SAPF and PAI on the level of anxiety and depression, a regression analysis was performed for all the subjects. The independent variables were SAPF and PAI from study II, staying at home, and leaving home now. Dependent variables were anxiety and depression.

The analysis showed that the model was statistically significant for both anxiety (R^2^ = 0.33; *p* < 0.05) and depression (R^2^ = 0.34; *p* < 0.05). A reduction of statistically insignificant variables was performed and a model explaining 30% odds for anxiety and 27% for depression was obtained. In both cases, SAPF remained the only independent variable (*p* < 0.01) after reduction.

## 4. Discussion

During the COVID-19 epidemic, the most important action for health care systems is to fight the pandemic directly. However, preventing the spread of the epidemic, consisting of a radical limitation of direct contacts and isolation, has and will have effects that require research, both in the psychological and behavioral sphere [13]. Psychological negative effects of isolation, consisting of increased stress, increased anxiety, and depression threats, were found in previous epidemics [14]. They concerned many social layers, people of different ages. However, the elderly and chronically ill, including PD patients, seem to be particularly at risk [15]. The reasons are mainly activity limitation, the related decrease in physical fitness, limited social contacts, often reduced treatment options, and the resulting increase in anxiety and depression [16].

The presented results of our research only show a statistically significant decrease in PAI for the total number of respondents, which is a natural consequence of isolation due to COVID-19. Similar observations were made by Shalash et al. [17]. It is a bad prognosis for the continued well-being and functioning of PD patients, as the beneficial effects of exercise on health are well documented [18,19]. This also applies to the relationship between physical fitness and health self-assessment [20]. The lack of differences between group A and group B indicates that the hypothesis about the relationship of activity, self-assessment of fitness, and mental condition with the way of running a household has not been confirmed. The reason for the lack of differences may be both the limitation of this study—a relatively small number of probes, and the individual lifestyle of the respondents. The importance of individual lifestyle is indicated both by the lack of differences between the groups of the remaining studied variables regarding behavior after isolation, as well as anxiety and depression. This is in line with our previous research [21]. Another limitation of this study is the lack of measurement of anxiety and depression in study I. Such a study was conducted by Otomani et al. They found no general differences in anxiety and depression after 6 weeks of isolation due to COVID-19 [22]. These authors also found no association of age, gender, and functional limitations associated with PD with both anxiety and depression. Both the results of the studies presented here and those of the Ottomans should be approached with caution due to cultural, environmental, and individual differences as well as the numerous risks associated with isolation due to COVID-19 [14]. One of the main risks was the inability to exercise with a therapist, which is particularly important in people with severe neurological disorders [23]. In the long run, this may negatively affect both the functional and mental states of patients [24].

Views on the frequency of anxiety and depression in PD patients are different. Previous research results ranged from 13.5% [25] to 40% [26]. Regardless of detailed and differing reports, this is a serious problem in PD patients. This is confirmed by the results of the qualitative assessment of these disorders presented here. Half of the respondents exceeded the normative values of anxiety and 60% of depression. Researchers do not agree as to the causes of these disorders, and the theory that they are a result of medical, neurochemical, and psychosocial phenomena has not yet been unequivocally confirmed empirically [27]. The presented results indicate that an important factor is the assessment of one’s own fitness. However, it does not fully explain all predictors of affective disorders. However, a decrease in the average SAPF in the second study may indicate that both the lack of physical activity and the psychological situation related to isolation and the risk of COVID-19, as well as reduced social contact, aggravate the severity of anxiety and depression. However, this requires further research due to the number of people studied here, which is a significant limitation.

## 5. Conclusions

Pandemic isolation has significantly reduced physical activity in PD patients. There was a certain drop in the self-esteem of physical fitness in these people. Physical fitness is an important predictor of preventing the affective disorders of anxiety and depression. The effects of isolation due to COVID-19 require further research.

## Figures and Tables

**Table 1:** Self-assessment of physical fitness and physical activity: Study I and Study II.

Variable	Group	Study I				Study II				I–II: *p* ^1^
		Median	Mean (SD)	±95% CI	A-B: *p*	Median	Mean (SD)	±95% CI	A-B: *p*	
SAPF	total	4.00	3.36 (1.18)	2.84–3.89		3.00	3.13 (1.14)	2.71–3.56		0.149
	A	4.00	4.00 (0.82)	2.70–5.30	0.300	4.00	3.76 (1.03)	2.58–4.75	0.219	1.000
	B	4.00	3.22 (1.22)	2.62–3.83		3.00	3.00 (1.14)	2.52–3.48		0.182
PAI	total	16.00	15.86 (4.90)	13.69–18.04		13.50	13.63 (3.81)	12.21–15.06		**0.034** *^2^
	A	16.50	17.25 (5.74)	8.12-26.38	0.712	14.50	14.83 (5.49)	9.07–20.60	0.494	0.479
	B	16.00	15.56 (4.83)	13.15-17.96		13.00	13.33 (3.36)	11.92–14.75		0.080

Abbreviations: SAPF, self-assessment of physical fitness; PAI, Physical Activity Index; A, people living alone; B, people living with a spouse or family; nss, not statistically significant; ^1^ with the continuity correction; ^2^ standardized effect, 0.739; power, 0.97; critical value, 2.05; *, statistically significant.

**Table 2:** Behavior and level of affective disorders—Study II.

Variable	Group	Median	Mean (SD)	±95% CI	A-B: *p*
Staying at home	total	30.50	42.33 (35.70)	29.00–55.66	
	A	20.50	34.50 (39.92)	−7.70–76.40	0.496
	B	30.50	44.29 (35.21)	29.42–59.16	
Leaving home before the epidemic	total	3.00	2.30 (.88)	1.97–2.63	
	A	3.00	2.67 (.82)	1.81–3.52	0.222
	B	2.50	2.21 (.88)	1.84–2.58	
Leaving home now	total	2.00	2.30 (1.18)	1.86–2.74	
	A	2.00	2.33 (1.37)	.90–3.77	0.978
	B	2.00	2.29 (1.16)	1.80–2.78	
HADS–anxiety	total	7.50	7.47 (4.21)	7.47–9.04	
	A	7.50	7.67 (3.20)	4.30–11.00	0.917
	B	7.50	7.42 (4.48)	5.52–9.31	
HADS–depression	total	8.00	7.97 (4.51)	6.28–9.65	
	A	9.50	8.33 (4.41)	3.70–13.00	0.603
	B	8.00	7.88 (4.62)	5.92–9.83	

Abbreviations: A, people living alone; B, people living with a spouse or family.

## Data Availability

Data are archived with the first author and in the Department of Neurology of the University Hospital where they were conducted. If necessary, contact the first author by e-mail aknapik@sum.edu.pl or www.katedrafizjoterapii.sum.edu.pl.
